# Cutting out the fat: Site-specific deacylation of an ion channel

**DOI:** 10.1074/jbc.H120.016490

**Published:** 2021-01-13

**Authors:** Pedro J. del Rivero Morfin, Manu Ben-Johny

**Affiliations:** Department of Physiology and Cellular Biophysics, Columbia University, New York, New York, USA

## Abstract

*S-*Acylation, a reversible post-translational lipid modification of proteins, controls the properties and function of various proteins, including ion channels. Large conductance Ca^2+^-activated potassium (BK) channels are *S-*acylated at two sites that impart distinct functional effects. Whereas the enzymes that attach lipid groups are known, the enzymes mediating lipid removal (*i.e.* deacylation) are largely unknown. Here, McClafferty *et al.* identify two enzymes, ABHD17a and ABHD17c, that excise BK channel lipid groups with remarkable precision. These findings lend insights into mechanisms that orchestrate the (de)acylation that fine-tunes ion channel function in physiology and disease.

Post-translational modifications (PTMs) of ion channels are fundamental to channel life cycle. By changing the chemical nature of specific amino acids, PTMs allow ion channels to respond to changes in cellular signaling by modifying channel structure, function, location and interacting partners. Ion channels and their subunits, which reside in a lipid-rich environment, are often modified by the covalent linkage of specific lipid groups (1), yet the mechanisms by which lipid modifications alter channel function, the identity of molecular players that orchestrate this regulation, and their overall physiological impact remain to be fully elucidated. Unlike other permanent lipid modifications, protein *S-*acylation—the intracellular addition of long-chain lipids, most frequently palmitate—is privileged, as it is the only known reversible lipid modification (1, 2). This unique feature makes *S-*acylation an attractive regulatory mechanism for ion channels.

Excitingly, a burgeoning list of ion channels and ion channel subunits have been identified as targets of *S-*acylation (1), including BK channels, voltage-gated sodium channels (3, 4), voltage-gated potassium channels, and voltage-gated calcium channel auxiliary β_2A_ subunit (5) among many others (1, 6). Like other PTMs, *S-*acylation tunes multiple facets of channel function, such as surface-membrane trafficking and channel activity (1). Importantly, distinct modes of functional regulation are imparted by *S-*acylation of disparate sites, suggesting sophisticated mechanisms of regulation much like other PTMs (7). Unlike other PTMs, the enzymes that orchestrate dynamic acylation and deacylation are largely unknown for most ion channels (2). Beyond this, it is also unknown whether these enzymes act with specificity, highlighting the need for in-depth mechanistic investigation of *S*-acylation.

The Shipston laboratory has been working to fill this gap in knowledge. They previously identified two distinct *S-*acylation sites in BK channels: 1) a motif within the S0–S1 linker that is intimately linked to channel trafficking and 2) a segment of an alternatively spliced stress-regulated exon (STREX) that enhances the apparent calcium sensitivity of the channel and influences channel regulation (1, 7). Interestingly, lipid attachment at the two sites are, in fact, catalyzed by distinct enzymes within the zinc-finger acyltransferases (zDHHCs) family (7). Furthermore, Lypla1 and Lyplal1, members of the classical lysophospholipase (LYPLA) family, have been shown to deacylate the S0–S1 linker (1, 7). However, it is unclear which enzymes mediate removal of lipid groups from STREX, as well as their target selectivity.

In this issue of JBC, a new study by Shipston and colleagues identifies α/β-hydrolase domain–containing protein 17 (ABHD17) as the protein acyl thioesterase that selectively removes palmitoylation of the STREX linker to tune channel gating (8). In this work, the authors devised a simple assay for probing the membrane localization of a GFP-tagged peptide containing the isolated STREX cysteine-rich domain in the presence of 21 candidate protein acyl thioesterases. In HEK293 cells, the GFP-tagged peptide is palmitoylated and is located at the intracellular surface of the plasma membrane. Remarkably, co-expression of either ABHD17a or ABHD17c dislodged the membrane-bound peptide. By contrast, Lypla1 and Lyplal1 had no effect on localization of the STREX cysteine-rich domain peptide. Using acyl-resin–assisted capture assays, the authors then probed the effect of ABHD17a/c on full-length BK channels by investigating palmitoylation of two channel variants: 1) a ZERO variant that lacks the STREX domain but has intact S0–S1 acylation and 2) a mutant channel containing the STREX domain but with S0–S1 palmitoylation sites disabled. Consistent with their hypothesis, STREX linker palmitoylation of the latter mutant was diminished with overexpression of ABHD1a/c. However, the ZERO variant was fully recalcitrant to both ABHD17a and ABHD17c. This finding is particularly exciting as there are no strict consensus sequences for palmitoylation (1), and it suggests that protein acyl thioesterases are not indiscriminately excising lipids; rather, they are highly selective and can distinguish between distinct acylation sites that may be closely juxtaposed within the same target.

This enzymatic selectivity also raised an important question: How does differential deacylation of the BK channel impact its function? To address this, the authors dissected the effect of ABHD17a and ABHD17c on membrane trafficking of BK channels compared with its activity at the plasma membrane. They quantified trafficking using a dual-labeling approach where channels at the surface membrane were specifically measured using antibodies that recognize an extracellular FLAG epitope, whereas the total amount of channels was determined using antibodies that detect a cytosolic hemagglutinin tag. Channel activity at the membrane was measured using an optical assay detecting membrane potential changes in response to Ca^2+^ influx via ionomycin. These experiments revealed no effect of ABHD17a/c on membrane trafficking but showed diminished magnitude of Ca^2+^-induced hyperpolarization. These findings point to an elegant mechanism of BK channel regulation by *S-*acylation (Fig. 1). The differential palmitoylation of the S0–S1 domains *versus* the STREX insert results in functional differences, wherein S0–S1 palmitoylation promotes membrane trafficking while STREX palmitoylation enhances channel activity. Conversely, Lypla1 and Lyplal1 mediate deacylation of the S0–S1 domain, whereas ABHD17a/c removes *S-*acylation of the STREX segment. In this manner, a rich collection of acyltransferases and acyl thioesterases orchestrate BK channel lipidation to ultimately tune channel activity.

**Figure 1:**
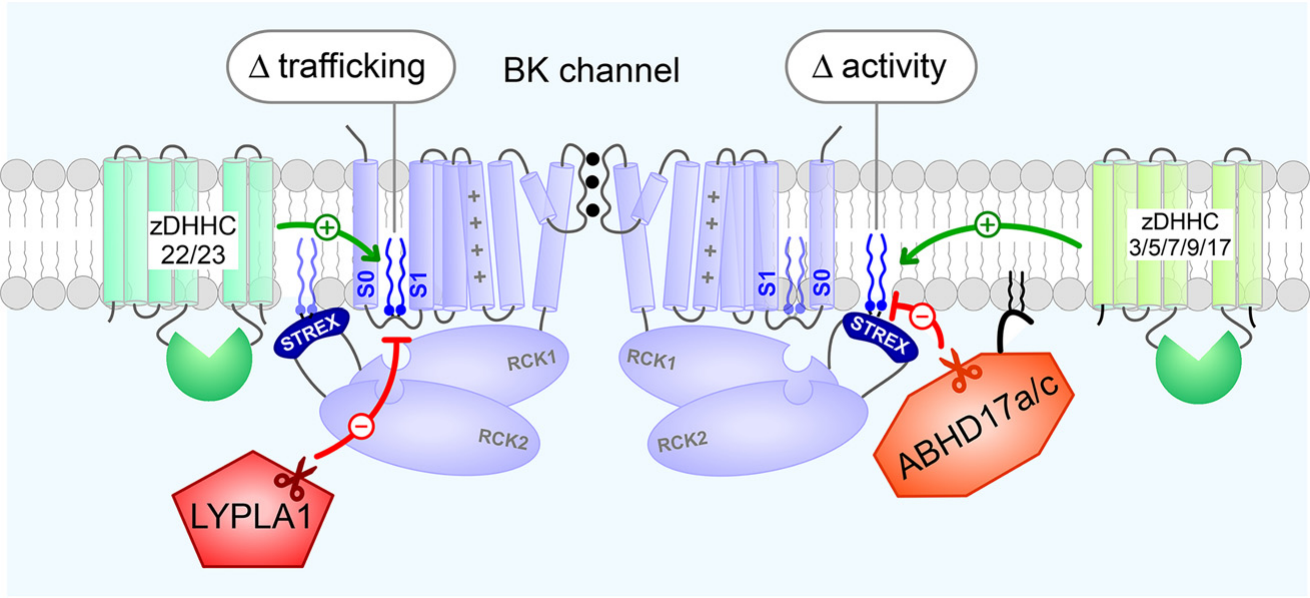
Schematic shows that site-specific *S-*acylation of BK channels orchestrated by distinct acyltransferases and protein acyl thioesterases evokes distinct functional effects.

In all, this study opens new frontiers in BK channel *S-*acylation and raises important questions. First, although the steady-state effects of BK channel *S-*acylation are now clear, the kinetics of such changes are still to be determined. Are there factors that either accelerate or decelerate this PTM in a physiological setting? Second, do subcellular differences in the *S-*acylation pattern of BK channels exist in neurons? For example, local palmitoylation and depalmitoylation are thought to affect post-synaptic density 95 (PSD95) within neuronal dendritic spines (6). Third, are there physiological factors that up-regulate or down-regulate activity of thioesterases? This study also bears important implications for other ion channels. For instance, cardiac voltage-gated Na^+^ channels are directly palmitoylated at multiple sites, and mutations in one particular site have been linked to cardiac arrhythmias (3). Similarly, palmitoylation of the β_2A_ subunit of voltage-gated Ca^2+^ channels fine-tunes channel inactivation and baseline activity. This lipid modification also competes with the phospholipid phosphatidylinositol 4,5-bisphosphate (9) and arachidonic acid (10), two downstream effects of G-protein–mediated signaling. Thus, are there depalmitoylating enzymes that dynamically and specifically reverse palmitoylation of the β_2A_ subunit to rapidly alter Ca^2+^ influx in certain physiological settings? The approach used by McClafferty *et al*. could assist in resolving these open questions, lending insights into pathophysiological mechanisms and helping to unravel the complex biology of *S*-acylation. An exciting era of biological discovery is on the horizon.

10.13039/100000065HHS | NIH | National Institute of Neurological Disorders and Stroke (NINDS) (R01 NS110672) to Manu Ben-Johny
